# Rethinking Breast Cancer Diagnosis through Deep Learning Based Image Recognition

**DOI:** 10.3390/s23042307

**Published:** 2023-02-19

**Authors:** Deawon Kwak, Jiwoo Choi, Sungjin Lee

**Affiliations:** 1 Electronic Engineering Department, Dong Seoul University, Seongnam 13120, Republic of Korea; 2 Choi’s Breast Clinic, 197, Gwongwang-ro, Paldal-gu, Suwon-si 16489, Republic of Korea

**Keywords:** breast cancer diagnosis, image classification, image segmentation

## Abstract

This paper explored techniques for diagnosing breast cancer using deep learning based medical image recognition. X-ray (Mammography) images, ultrasound images, and histopathology images are used to improve the accuracy of the process by diagnosing breast cancer classification and by inferring their affected location. For this goal, the image recognition application strategies for the maximal diagnosis accuracy in each medical image data are investigated in terms of various image classification (VGGNet19, ResNet50, DenseNet121, EfficietNet v2), image segmentation (UNet, ResUNet++, DeepLab v3), and related loss functions (binary cross entropy, dice Loss, Tversky loss), and data augmentation. As a result of evaluations through the presented methods, when using filter-based data augmentation, ResNet50 showed the best performance in image classification, and UNet showed the best performance in both X-ray image and ultrasound image as image segmentation. When applying the proposed image recognition strategies for the maximal diagnosis accuracy in each medical image data, the accuracy can be improved by 33.3% in image segmentation in X-ray images, 29.9% in image segmentation in ultrasound images, and 22.8% in image classification in histopathology images.

## 1. Introduction

The AI (Artificial Intelligence) is being applied to various industries thanks to the recent great success [1,2,3,4]. In particular, the performance improvement is remarkable on the area of image recognition technologies such as image classification, object detection [5] and image segmentation [6,7], so that these technologies are enormously being applied to various industrial applications such as autonomous driving, robots, meta-verse, industrial automation, smart city, medical care and healthcare.

Above all, the medical imaging diagnosis technology though the images obtained from medical imaging devices (ultrasound, MRI, CT, X-ray and Endoscope) is being applied into the domains of trained medical professionals such as gastrointestinal diseases detection [8,9,10], heart disease diagnosis [11,12], tumor detection [13,14] and so on. Moreover, the artificial intelligence in the medical industry are also being applied to the territory of new drug development [15,16]. In the future, it is expected that the medical and healthcare industry will face a technological renaissance thanks to the artificial intelligence.

However, because building a large-scale dataset, which is one of key components for AI based medical technology, requires a lot of cost and even contains sensitive private information, the dataset has not been provided sufficiently to encourage the development of artificial intelligence. Nevertheless, as research using medical artificial intelligence has recently been actively conducted not only in academia and industry, but also in the government, datasets are being built through large-scale investments, and related laws are also being revised.

For this goal, the image recognition technologies for optimizing the diagnosis process using each medical image data are investigated in terms of various image classification techniques (VGGNet19 [1], ResNet50 [2], DenseNet121 [3], and EfficientNet v2 [4]), image segmentation techniques (U-Net [17], ResU-Net++ [18], and DeepLabV3 [19]), and related loss functions (Binary Cross Entropy, Dice Loss, Tversky Loss), and data augmentation techniques.

This paper studied a technique for diagnosing breast cancer using artificial intelligence based medical image recognition technology. Therefore, mammography (examination of human breast using low energy X-ray) images, ultrasound images, and histopathology (microscopic examination of tissue) images are used to improve the accuracy of the process of diagnosing breast cancer classification through image classification technique and of inferring their affected location through image segmentation technique. Then, the image recognition application strategies for optimizing the diagnosis process using each medical image data are investigated in terms of various image classification techniques (VGGNet19 [1], ResNet50 [2], DenseNet121 [3], and EfficientNet v2 [4]), image segmentation techniques (U-Net [17], ResU-Net++ [18], and DeepLabV3 [19]), and related loss functions (Binary Cross Entropy, Dice Loss, Tversky Loss), and data augmentation techniques.

## 2. Related Work

The authors of [20] studied the data augmentation in the image classification task using X-ray images in general. In particular, The types and characteristics of available datasets are summarized. The authors of [21] analyzed the X-ray image dataset to improve the accuracy of image classification, and in particular, investigated the data augmentation methods in medical datasets. The study of [22] dealt with the data augmentation techniques in the field of CWP (Coal Workers’ Pneumoconiosis) using X-ray. However, X-ray image recognition researches in one medical domain [21,22] may have performance limitations in direct application to another medical domain such as breast cancer diagnosis, so that additional verification is requested according to the domain change.

For the mammography dataset, the authors of [23] conducted various studies on data augmentation methods (translation, rotation, scaling, flipping, resizing, pixel level augmentation, pseudo-colour augmentation, random erasing, kernel filter, GAN based augmentation, style transfer) for image classification in X-ray images. However, any performance evaluation result was not presented.

The authors of [24,25,26,27,28,29,30] represented the performance for breast cancer classification in mammography images by using traditional data augmentation based on noise, Gaussian filter, jitter, scale, powers, rotate, shear and flips. According to the result of [24], Gaussian filter shows the best accuracy (0.881) compared to the others, i.e., Rotate (0.880), Shear (0.879). On other hand, the authors [25] claim the all combinations of rotation, mirroring, Poisson noise and RoI extraction increase the accuracy of 71% into 76.2%. The result of [26] presents the data augmentation effect of 95.7% from 87%. The authors of [29] claim that the data augmentation has the effect of 20∼30% enhancement in accuracy.

In study of [31], various segmentation techniques using breast cancer diagnostic images were classified into traditional methods (region, threshold, edge methods) and machine learning methods (supervised, unsupervised, and deep learning methods). For X-ray image segmentation, it was proved that the deep learning methods are more advantageous than the traditional methods because they do not require pre- and post-processings.

In this paper, it is investigated how the medical devices used for breast cancer diagnosis, i.e., X-ray (Mammography), Ultrasound, Histopathology and MRI (Magnatic Resonance Imaging), are used in the diagnosis process. In particular, appropriate image recognition technologies were derived based on the characteristics of each medical procedure for X-ray, ultrasound, and pathology images mainly used in general hospitals. In addition, the performance was evaluated based on the various combinations of loss functions, neural network models and data augmentation methods on the open medical datasets [32,33,34]. Finally, the optimal combination application strategies for each medical device were presented.

## 3. Breast Cancer Diagnosis Process and Related Image Recognition Technology

### 3.1. Breast Cancer Diagnosis Process

Before proposing a deep learning-based breast cancer diagnosis technology, the basic process of diagnosing the breast cancer by doctors is introduced with related technical discussion in each process.

**Figure 1:**
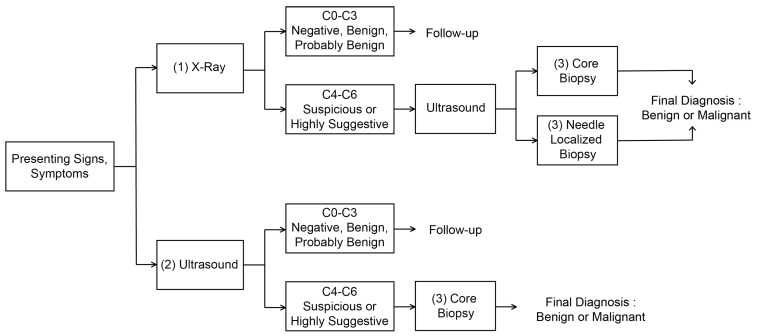
Process of breast cancer diagnosis.

Figure 1 shows the breast cancer diagnosis process. First, the breast cancer can be categorized into two types: The first is a breast cancer incidence type with microcalcification, and the second is a breast cancer incidence type that occurs as a lump. Due to their unique physical characteristics, they can only be discovered by utilizing different imaging methods, that is, X-ray and ultrasound examinations. However, all of these diagnostic imaging methods cannot be accurate diagnosis and are only a step of making predictions. For an accurate diagnosis, a histopathological examination step through the biopsy based on the location prediction of the breast disease lesion should follow. Let us summarize the whole procedure as shown in Figure 1 as follows:

1. **Diagnosis of breast cancer with microcalcification (possibility of breast cancer) and prediction of suspected lesion through X-ray examination:** Mammography is the process of examining the breast of patients for diagnosis and screening using low-energy X-rays (usually about 30 kVp). The goal of mammography is to detect breast cancer early, usually through the detection of characteristic masses or microcalcifications [35]. In particular, the mammography inspection can easily detect the breast cancer accompanied by microcalcification due to its physical characteristics. Since the microcalcification is difficult to be detected in ultrasound imaging, it should be diagnosed through X-ray examination. The test performs a diagnosis of the condition about the affected area. In addition, the location of the suspected area is also predicted through more diverse data through longitudinal and transverse X-ray examination of the affected area. This can also be inaccurate depending on the size and condition of the suspected area, which means that only the possibility of breast cancer and tumor development can be determined. The result of the diagnosis prediction can be obtained based on BI-RADS Category 0–6 [36] depending on the severity of symptoms, so that subsequent procedures vary according to these results. Among these, if a result corresponding to BI-RADS Cat0-3 is shown, that is, in the case of negative, benign, or possibly benign (Probably Benign), a follow-up reexamination should be proceeded after about 3–6 months. If malignant, the result corresponding to BI-RADS Cat4-6, is suspected, ultrasound examination should be performed to confirm the result, and the final diagnosis about the malignant is made by proceeding with (3) Core Biopsy or Needle Localized Biopsy.
2. **Diagnosis of lump-type breast cancer (possibility of breast cancer occurrence), location diagnosis and prediction of suspected area through ultrasound examination:** In addition to the breast cancer with microcalcification, lump-type breast cancer can be detected by the ultrasound image due to its physical characteristics. Ultrasound images are variable and inaccurate in shape, making it difficult to discern even by professionals. Therefore, professionals sometimes record ultrasound images of the affected area and view them again. This ultrasound examination is different from the ultrasound examination for confirmation of X-ray examination mentioned in the previous step (1). This ultrasound examination confirms the result based on BI-RADS Cat0-6 by diagnosing the affected area regardless of the X-ray examination result. If the results of BI-RADS Cat0-3 are confirmed, a follow-up re-examination should be proceeded after about 3 months in the same way as in the (1) X-ray inspection step. If malignant, the result corresponding to BI-RADS Cat4-6, is suspected, a histopathology test should be performed through Core Biopsy, which is the step (3) to confirm the result, to determine the final diagnosis of the malignant. In addition, Needle Localized Biopsy is not performed in this (2) ultrasound examination stage, because Needle Localized Biopsy is a step to discover lesions that can only be found in X-ray examination.
3. **Precise diagnosis through histopathology of suspicious parts:** If the probability of malignancy is high in the previous step, a biopsy of the suspected area and its histopathological examination should be performed. Here, the pathological examination is performed by slicing some tissues obtained from the biopsy of the suspicious area. As long as the suspected area is properly collected at this stage, it is a test with the lowest probability of error in breast cancer diagnosis, so that it is possible to determine the presence or absence of malignancy quite accurately. However, since the diagnosis of breast cancer can be inaccurate if tissues are not collected for the suspected area, the diagnosis prediction and location prediction in the previous steps (1)–(2) must be accurately made. If the diagnosis of cancer is correctly made at this stage, treatments can be performed.
4. **Additional examination and surgery:** In fact, previous step (3) is the end of diagnosis. However, if the surgery should be operated among the aforementioned treatments, MRI examination should be additionally performed for checking the size of breast cancer lesion. Then, surgey is operated for the stage diagnosis and removal of the suspected lesion. Mammography could be not enough for young women with dense breasts, because lack of functional information makes it difficult to detect lesions. Ultrasound scans can detect tumors in women with dense breasts. It helps to characterize, size, and location tumors [37] However, ultrasound scans are also ineffective in detecting early lesions of microcalcification. MRI is currently being used to complement mammography in breast lesion diagnosis. MRI utilizes magnetic fields to generate detailed cross-sectional images of tumor structures, providing superior quasi-soft tissue contrast. Breast tissue, fat, and tumors appear differently in images. MRI scans with intravenous injections of contrast media can more reliably detect mutations [38]. The sensitivities and negative predictive values (NPVs) were significantly higher for MRI compared with mammography for detecting breast cancer (98.4% vs. 89.8% and 87.8% vs. 46.6%, respectively) [39]. MRI cannot completely replace mammography and ultrasound scan, but it is a means to complement them because it is expensive, time consuming and uncomfortable for claustrophobic patients. Lastly, because there is a possibility that undiscovered small malignant tissues exist around the lesion, the surgery is performed by excising up to the periphery (normally, 3–5 cm) of the confirmed lesion in practice.

As can be seen through the previous breast cancer diagnosis process, the most final and accurate diagnosis is the histopathological examination in step (3). However, if the lesion location predicted in steps (1) and (2) is incorrect, the histopathlological examination in step (3) could cause incorrect diagnosis. In addition, for the correct prediction of breast cancer lesion, the repetition of steps (1) and (2) prior to the steps (3) may cause patients to feel burdened and stressed, so that an accurate diagnosis is recommended in the preceding (1) and (2) steps to avoid unnecessary stress and cost increases for these patients. From the perspective of most hospitals and from the perspective of patients diagnosed through regular inspection, it is possible to avoid the costs and unnecessary stress on patients by maximizing the diagnosis accuracy of stages (1)–(3). The development of deep learning image recognition technology can be an important means of increasing the diagnostic accuracy in the above steps (1)–(3), so that it can reduce the pain and cost of patients from the repetition of unnecessary inspections.

### 3.2. Image Recognition Technology Related to Breast Cancer Imaging Diagnosis

As seen in the above steps, not only cancer diagnosis but also identification of the location of the affected area play an important role in the stages of (1) X-ray examination and (2) ultrasound examination. On the other hand, in the pathology diagnosis stage of step (3), because the suspicious area has already been identified with the collected biopsy tissues, diagnosis becomes more crucial than the identification of the location. Therefore, this study verifies the performance and establishes the optimization strategies by applying the image recognition technologies according to each medical image data as shown in Table 1.

For the evaluation, the detailed models in image segmentation and image classification were selected based on the performance and the number of citations [17,18,19]. The applied datasets were based on the most usable datasets among the open datasets [32,33,34]. The next subsections briefly introduce these datasets, detailed image recognition technologies for diagnosing them, and applied data augmentation methods.

#### 3.2.1. Evaluation Dataset

As a mammography dataset, this paper utilizes the CBIS-DDSM dataset [32]. This dataset includes three types of mammography images as the tumor information of 1566 patients, i.e., benign tumor, malignant tumor, and normal data. There are about 4090 benign images, 4090 malignant images, and 2040 normal images, totaling 10,239 images.

The BUI (Breast Ultrasound Images) was utilized as the ultrasound image dataset [33]. This dataset determines whether breast ultrasound images contain early signs of breast cancer tumors and microcalcification. There are 891 benign images, 421 malignant images, and 266 normal images for a total of 1578 images.

The BHI (Breast Histopathology Images) [34] was utilized as the pathology image dataset. This dataset determines whether or not IDC (Invasive Ductal Carcinoma) is included in a breast pathology image. There are 198,738 benign images and 78,786 malignant images for a total of 277,524 images.

**Figure 2:**
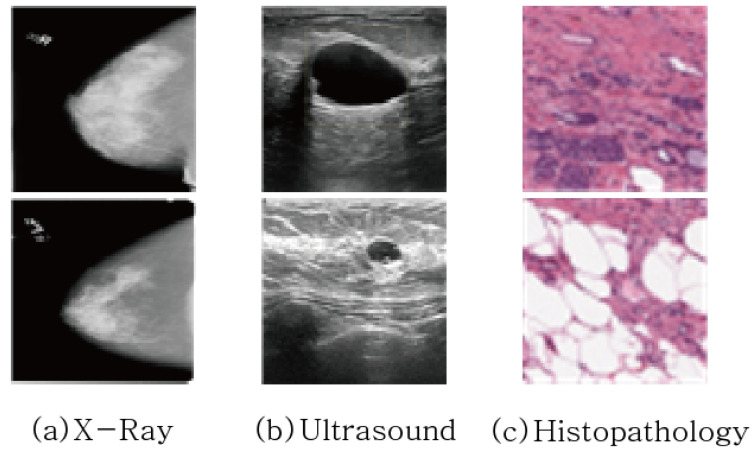
Example of Medical Image Data used for Breast Cancer Diagnosis.

Figure 2 shows examples of three medical image datasets. From Figure 2, it can be observed that the class imbalance between the foreground, i.e., the tumor part, and the background, i.e., non-tumor part, is quite significant. Statistically, the ratios of background and foreground are 99.56:0.44 in X-ray dataset and 85.5:14.5 in ultrasound dataset. The class imbalance problem affects segmentation performance, so that it will be overcome by the exploration of various loss functions in this paper.

#### 3.2.2. Image Classification Model

ILSVRC’s ImageNet dataset is a large-scale dataset containing 1000 classes and 1.2 million training images. Image Classification networks, which are designed to be optimized for such large-scale datasets, have been being designed deeper to have higher accuracy. However, when these networks are operated on datasets with a small number of classes in most of application scenarios, a serious over-fitting phenomenon occurs [40]. Therefore, in the image classification problem for breast cancer diagnosis targeted in this study, the total number of classes is two (benign, malignant), and the total number of images is only hundreds to tens of thousands, so that an excessively deep network could occur overfitting and the performance could deteriorate. Therefore, in this study, performance verification was performed focusing on VGGNet19 [1], ResNet50 [2], DenseNet121 [3], and EfficientNet v2 [4], which have excellent accuracy performance with relatively low number of layers.

Here, let us briefly examine the architectures of the considered image classification models. At early stage of deep learning research, main concern was to design the network into more deeper structure with avoiding performance degradation. Therefore, the authors of VGGNet [1] were trying to increase depth of an architecture with very small (3 × 3, 1 × 1) convolution filters instead of using large filters (5 × 5, 7 × 7), which showed a significant improvement by increasing the depth to 16–19 weight layers. On the other hand, the authors of ResNet [2] were trying to solve the vanishing gradient problem accrued from designing deeper network by introducing residual learning block (shortcut connection), which achieved 3.57% error on the ImageNet test set with the depth of up to 152 layers. Last, as the extended version of ResNet, DenseNet [3] proposed the architecture in which each layer connects to all other layers in a feed forward path. Unlike ResNet, where each layer has a connection structure with the next lower layer, DenseNet proposed a new structure with L(L+1)/2 direct connections, which showed more performance improvement in ImageNet test set than the previous ResNet.

#### 3.2.3. Image Segmentation Model

U-Net [17], ResU-Net++ [18], and DeepLabV3 [19], which are the most widely known image segmentation networks for X-ray image and ultrasound image diagnosis, are used to verify and optimize performance.

Here, let us present brief summary of each model. First, U-Net is an end-to-end FCN (Fully Convolutional Network) based model proposed for image segmentation in the biomedical field. It consists of a contracting path, which is a network to capture context and a symmetric expanding path that enables precise localization [17]. In particular, the expanding path outputs a segmentation map by using feature maps from intermediate layers of the contracting path. The structure of the contracting and expanding paths is also called “encoder-decoder” structure. In addition, ResU-Net++, the extended version of ResU-Net, is configured by adding Residual Blocks, Squeeze and Excitation Block, ASPP (Atrous Spatial Pyramid Pooling), and Attention block technologies to the previous version. Last, DeepLabV3 is a method of obtaining a more dense feature map by using Atrous Convolution on the existing ResNet structure.

## 4. Data Augmentation Technique for Breast Cancer Imaging Diagnosis

In order to evaluate the effect of data augmentation in medical images, we set up the datasets through various data augmentation techniques. The data augmentation techniques to be considered are divided into Geometric and Filter methods and are shown in Table 2. As the geometric transformation, following schemes are utilized: Rotation, vertical flip, horizontal flip, grid distortion. In addition, the filter based augmentation employed in this paper are CLAHE, Gaussian Blur, equalization, fancy PCA. CLAHE (Constrast Limited Adaptive Histogram Equalization) is a variation of Adaptive Histogram Equalization that improve contrast in images by limiting the amplification [41]. Gaussian Blur is the result of blurring an image by a Gaussian function [42], Equalization is a method of constrast adjustment using the image’s histogram [43] Fancy PCA alters the intensities of the RGB channels along the natural variations of the images, denoted by the principal components of the pixel colors [44].

**Figure 3:**
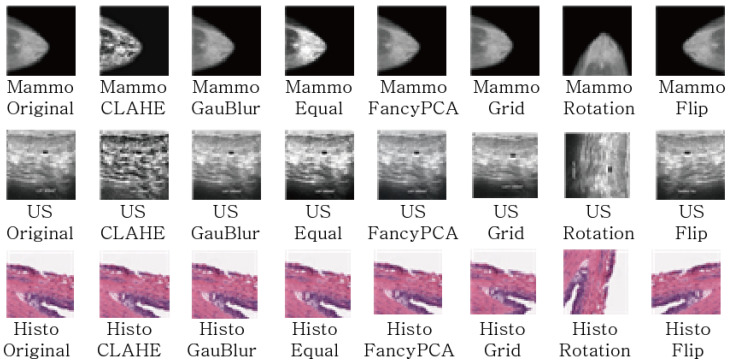
The Examples of Data Argumentation Techniques.

Figure 3 shows examples of image augmentation techniques applied to the three types of medical image data mentioned above.

**Figure 4:**
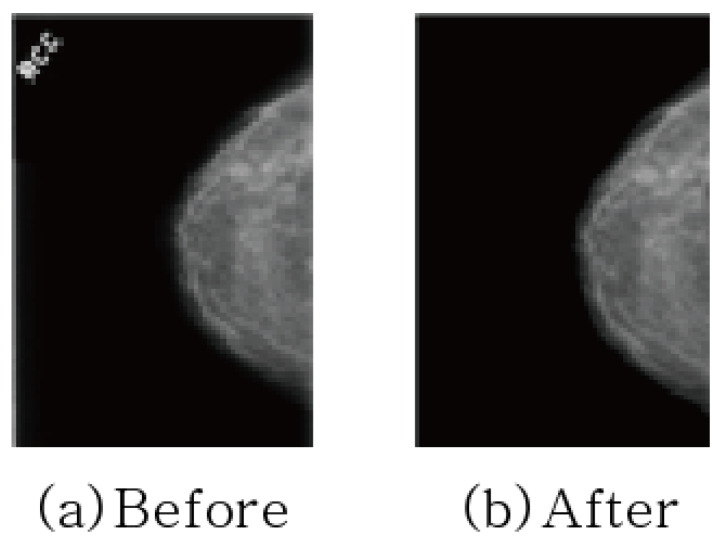
Removing texts in mammography.

In addition, text is sometimes included in mammography images to indicate direction. Since image features not related to learning can adversely affect learning, preprocessing was performed to erase text for all datasets as shown in Figure 4.

## 5. Loss Function for Segmentation

Then, let us examine the loss functions optimized for the medical image segmentation performance.

**Figure 5:**
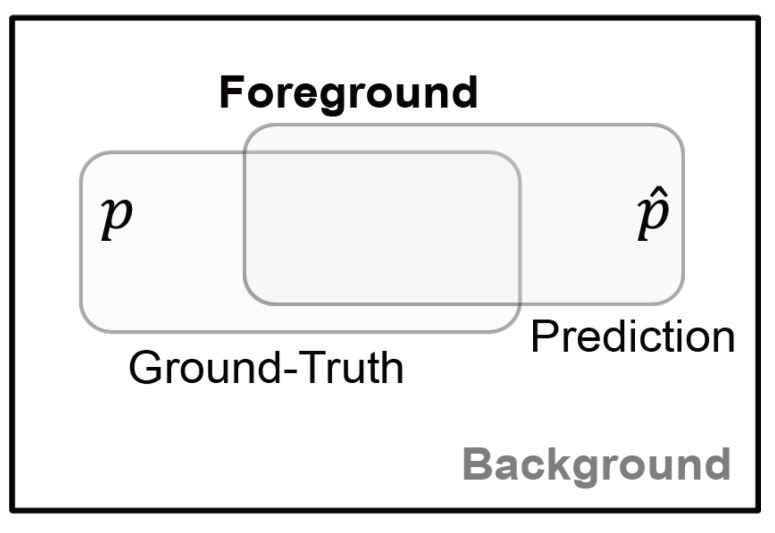
Parameters used for image segmentation.

In X-ray and ultrasound images, there are a total of two foreground classes (benign and malignant) and a background class. However, since the two foreground classes do not exist at the same time, the foreground class is denoted by a single parameter. To derive these mathematical functions, the following two parameters were defined as shown in Figure 5,

- *y*: the ground-truth of Foreground class lesion,
- $\hat{y}$: the prediction (probability) of the ground-truth *y*.

### 5.1. Binary Cross-Entropy

Binary Cross-Entropy is a function that measures the difference in probability distribution of two random variables is widely used in image classification and also in image segmentation by extending it to pixel unit classification. For the medical image segmentation of this paper, two classes, the foreground (benign or malignant) and the background, are classified with the probability value between 0 and 1.

(1) $$\begin{array}{ccc} L_{BCE}\left(y,\hat{y}\right) & = & -y\log\left(\hat{y}\right)+\left(1-y\right)\log\left(1-\hat{y}\right) \end{array}$$

### 5.2. Dice

It is a loss function commonly used in image segmentation and is derived from the dice coefficient that calculates the similarity between two images. The dice coefficient is designed to have a strength in an imbalanced state between classes, and the neural network is trained by placing a higher weight on the foreground class than on the background class.

(2) $$\begin{array}{ccc} L_{D}\left(y,\hat{y}\right) & = & 1-\frac{2y\hat{y}+1}{y+\hat{y}+1} \end{array}$$

### 5.3. Tversky

The Tversky loss was further developed dice loss based on statistical characteristics. The class imbalance problem results in high-precision and low-recall performance in image segmentation. In order to solve this problem, a weight that gives a penalty is newly introduced, so that the higher the value, the higher the recall. In particular, it becomes equal to the dice loss when α=β=0.5.

(3) $$\begin{array}{ccc} L_{T}\left(y,\hat{y}\right) & = & 1-\frac{1+y\hat{y}}{1+y\hat{y}+\beta \left(1-y\right)\hat{y}+\alpha y\left(1-\hat{y}\right)} \end{array}$$

## 6. Simulation Results

**Figure 6:**
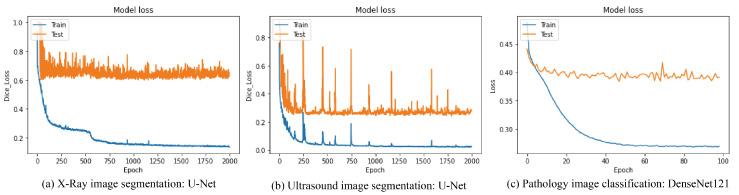
Training Procedures of each operation.

The accuracy of image segmentation and image classification for three types of medical image data as mentioned in Section 3.1 was evaluated in Table 3, Table 4, Table 5, Table 6, Table 7, Table 8 and Table 9 according to the loss functions, the neural network models and the data augmentation techniques. The training procedures of each operation are shown in Figure 6, which were trained up to the point before overfitting.

In addition, all experiments are performed on a server with specifications of I9-10980EX CPU, NVIDIA RTX 3090 2way and 128GB RAM. Moreover, the transfer learning based on the ImageNet is only adopted in the image classification task of pathology data. Lastly, remarks and strategies for performance optimization are derived based on the evaluation.

### 6.1. Prediction of Breast Cancer Diagnosis Through X-ray Images

**Figure 7:**
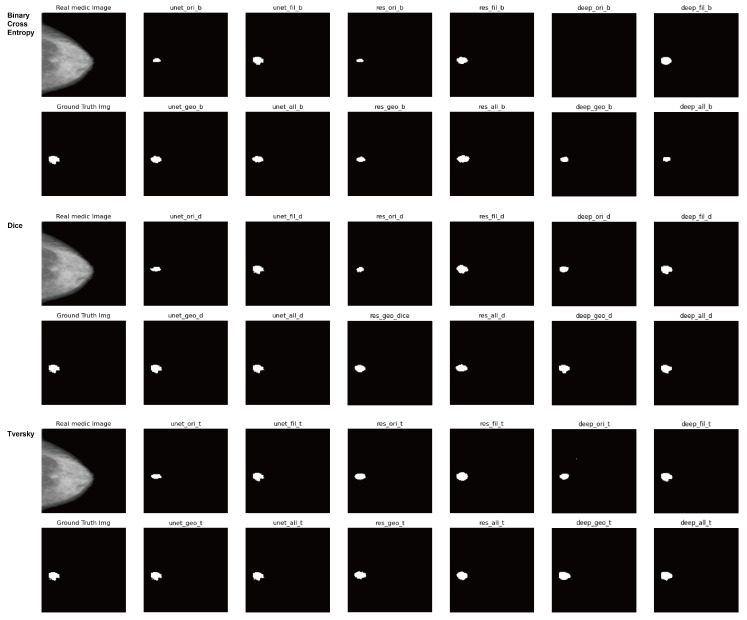
Result images for all combinations of the loss functions and the neural networks and the augmentation techniques in X-ray datasets.

As the results of diagnosing the breast cancer incidence type with microcalcification through X-ray examination of step (1) as mentioned in Section 3.1, Table 3 and Table 4 show the segmentation accuracy in terms of the loss functions and the data augmentation techniques respectively. Lastly, Figure 7 shows the result images for all combinations of the loss functions and the neural networks and the augmentation techniques.

First, an experiment was conducted to determine which loss function is preferred for training the X-ray image segmentation neural network. The experiments were performed on U-Net [17], ResU-Net++ [18], and DeepLabV3 [19] on the basic training dataset to which data augmentation was not applied. Also, the weight of the Tversky loss function was set to α=0.7, β=0.3.

As can be seen from the experimental results in Table 3, the dice loss and the Tversky loss show superior performance compared to the binary cross entropy loss in the two accuracy indicators, MeanIOU and DiceCoeff.

Due to the characteristics of this medical dataset that detects small-sized cancer tumor tissue, the class imbalance between foreground and background is significant, so that the Tversky loss and dice loss are preferable.

This is superior in performance to binary cross entropy, which is relatively advantageous for equal class distribution, and if weights are set optimally for a specific dataset, the Tversky loss can produce better performance than dice loss.

However, even if the weights of dice loss are not optimally set, performance almost close to the optimally set Tversky loss can be obtained. In other words, if there is little variation in the characteristics of the foreground/background class of the dataset, setting and using the weight of the Tversky loss accordingly can improve performance, but in reality, because the size of cancer tissue per data is very different (In fact, the size of the tumor varies depending on the degree of cancer progression and the imaging method), so it can be said that it is practically more appropriate to use the dice Loss, which shows better performance in normal cases, than to set and use it optimally for certain specific data.

**Remark 1.**  *The loss function to achieve the best performance in the medical X-ray image segmentation area is selected in consideration of the class imbalance of the data.*

Now, based on the dice loss and Tversky loss functions, we experimentally verify the optimal combination of various image segmentation techniques and data augmentation methods.

The data augmentation method used in this experiment is a geometric method (random rotation, vertical flip, horizontal flip, grid distortion), which directly changes the shape of the image. The filter-based method (Gaussian filter, image histogram equlization, CLAHE, fancy PCA) utilizes a data augmentation technique in which the characteristics of an image stand out by adding or removing noise using a filter or by changing RGB values of pixels. The number of basic vanilla dataset is 3185 images. The numbers of the geometric and filter datasets are augmented to 15,921 and 15,921, respectively.

As shown in Table 4, when using the two data augmentation methods in all neural network cases, the performance increases by up to 33.3% (IOU). This is because it reduces the risk of overfitting to small amount of data by increasing the number of data though the aforementioned augmentation, thereby helping to show more generalized performance. In particular, the filter-based data augmentation method shows greater performance improvement than the geometric-based data augmentation method. Even the dataset using only one filter method shows better performance than the method combining the filter method and the geometric method. This is because, due to the nature of medical data, the position and pose at the time of imaging the affected part are almost fixed, so the validity is low. On the other hand, it can be seen that the filter based augmentation shows a greater performance improvement, because it can be learned with more difficult data, i.e., the blurred image for the small size of the tumor.

On the other hand, in the case of neural network selection, ResUNet++ [18] has the best performance in the original dataset (Vanilla), while the U-Net [17] network has the best performance in the augmented dataset (Filter). Since ResUNet++ [18] or DeepLab V3 [19], which have a more complex structure than UNet [17], can be considered to have their own data augmentation function by creating various channels for the input image, the effect of additional data augmentation techniques is not so great. Also, in general, ResUNet++ [18] performs better than UNet [17] on large datasets. However, since the target medical dataset has a small number of classes and a small number of images, it can be seen that the optimal neural network is determined differently from general large-capacity datasets.

Figure 7 shows the result images for all combinations of the loss functions and the neural networks and the augmentation techniques.

Lastly, Table 5 shows the average time taken for each segmentation model. As shown in the results, UNet with the simple structure shows the least latency, and ResUNet++ and DeepLabV3 with complex structures take more latency. Through this, it can be seen that UNet is most suitable for X-ray segmentation task in terms of both performance and cost.

### 6.2. Prediction of Breast Cancer Diagnosis Through Ultrasound Imaging

**Figure 8:**
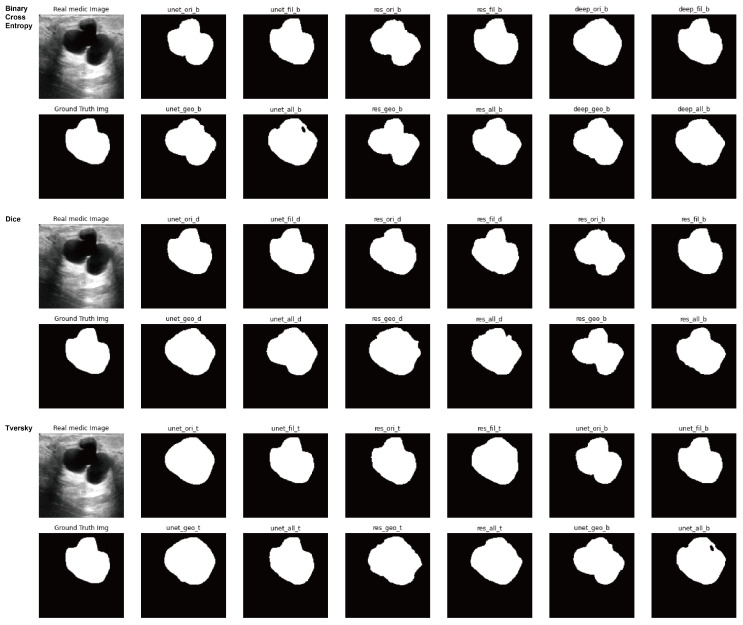
Result images for all combinations of the loss functions and the neural networks and the augmentation techniques in ultrasound datasets.

In order to optimally predict the occurrence possibility and the suspected location of lump-type breast cancer through the ultrasound examination of step (2) mentioned in Section 2, the optimal combination of the loss functions, the segmentation models and the data augmentation methods is investigated with their evaluation. Lastly, Figure 8 shows the result images for all combinations of the loss functions and the neural networks and the augmentation techniques.

Table 6 was evaluated to determine which loss function would be suitable for training the image segmentation neural network. Experiments were conducted on UNet [17], ResUnet++ [18], and Deeplab v3 [19] on the basic training dataset in the same experimental environment as X-ray. Also, the weights of the Tversky loss function were set to α=0.7 and β=0.3. As shown in Table 6, the dice loss function and the Tversky function have superior performance compared to the binary cross entropy loss in the two accuracy indicators, MeanIOU and DiceCoeff, as in the case of X-ray images. Moreover, when considering the characteristics of this medical data set to detect small-sized cancer tumor tissue, the dice loss or Tversky loss function for the class imbalance problem has advantages over binary cross entropy.

However, the Tversky loss theoretically should be better than dice loss in terms of performance by adding a degree of freedom for weight adjustment. However, as shown in the case of ResUNet++ [18] and DeepLab v3 [19] in Table 6, it should be noted that the performance advantage may vary because the optimal weight varies depending on the nature of the segmentation neural network.

**Remark 2.**  *The selection of the loss function to achieve the best performance in the medical image segmentation area must be determined in consideration of the neural network.*

Now, based on the dice loss and Tversky loss functions in ultrasound medical images, we experimentally verify the optimal combination of various image segmentation techniques and data augmentation methods. The data propagation method used in this experiment is the same as the geometric method and filter method of the previous X-ray method. The basic vanilla method was 1568 images, and the geometric and filter types were multiplied by 7840 and 7840 images. As known in Table 7, similar with the result of X-ray, the filter-based data augmentation method shows a greater performance improvement than the geometric-based data augmentation method, and shows a maximum of 29.9% (IOU) improvement. That is because it can be learned with more difficult data than the data whose borderline is blurred.

On the other hand, in the case of network selection, DeepLab V3 [19] is the best in the original dataset (Vanilla), and the U-Net [17] network shows the best performance in the data augmented dataset (Filter). Similar with the result of X-ray, since DeepLab V3 [19] with a more complex structure than UNet [17] can be considered to have their own data augmentation function by creating various channels for the input image, the effect of additional data augmentation techniques is not so great.

It can also be seen that in general setups, DeepLab V3 performs better than UNet, and for small ultrasound medical datasets, neural networks that look like normal contract sets are calculated.

**Remark 3.**  *In medical X-ray and ultrasound image segmentation, it is desirable to use a filter-based data proliferation method and select a neural network in consideration of the size of the data.*

Lastly, Table 8 shows the average time taken for each segmentation model in case of ultrasound image. According to the results, UNet with the simple structure shows the least latency, and ResUNet++ and DeepLabV3 with complex structures take more latency, which is same result as the case of X-ray segmentation. Through this, it can be seen that UNet is most suitable for ultrasound segmentation task in terms of both performance and cost.

### 6.3. Diagnosis of Breast Cancer Through Pathology Imaging

In this experiment, in order to maximize the accuracy of histopathology image classification, we evaluate the combinations of the image classification networks, (VGGNet19, DenseNet121, ResNet50, EfficientNet) and the data augmentation methods (Vanilla, MixUp, CutMix, Filter, GeoMetric). In addition, other reliability metrics, i.e., AUC (Area Under the Curve), SEN (Sensitivity) and SPE (Specificity), are evaluated as well. Here, Vanilla is the original image set and the data augmentation method of GeoMetric means the rotation, the flip, the transfer, the shear, the gaussian, the equalization and the shift as mentioned in Section 4.

The results in Table 9 and Table 10 show that ResNet50 has the best accuracy and reliability in all basic as well as all related augmented datasets than other neural networks, VGGNet19, DenseNet121, and EfficientNet. It can be seen that the required neural network capacity of the histopathology dataset is closest to ResNet50 among the aforementioned datasets. Even though DenseNet121 and EfficientNet show better performance than ResNet50 in ImageNet, they have lower performance than ResNet50 due to overfitting in this histopathology dataset. From this observation, it can be seen that the characteristics and capacity of the data act as important factors in selecting the optimal neural network for the small medical dataset. In addition, as can be seen from the results of Table 6, it is observed that the performance increases by up to 22.8% if the data augmentation method is used in all neural networks. This is because it helps to have more generalized performance by reducing the risk of overfitting to the small dataset by applying various variations to the limited size of the data set. In particular, as in the results of image segmentation, it can be seen that the filter-based method shows the best performance in image classification.

**Remark 4.**  *In medical breast pathology image classification, it is desirable to use a filter-based data augmentation method and select a neural network in consideration of the required capacity of the dataset.*

Lastly, Table 11 shows the average time taken for each image classification model of pathology image. According to the results, VGGNet19 and ResNet50 with simple structure shows less latency, and DenseNet121 and EfficientNet with complex structures take more latency. However, when considering the best performances of each classification model in Table 9, DenseNet121 and EfficientNet have weaknesses compared to ResNet50 in terms of both accuracy and cost. On the other hand, VGGNet19 has its own strength compared to ResNet50 in terms of cost. Nevertheless, considering that accuracy is more crucial point than cost in medical industry, ResNet50 is most recommended among the aforementioned models for the medical breast pathology image classification task in terms of both performance and cost.

## 7. Conclusions

In this paper, we examine how each procedure is used in the diagnosis process using all medical device images used for breast cancer diagnosis, that is, X-ray (Mammography), ultrasound, and pathology images (Histopathology). Based on the procedural features, suitable image recognition technologies were derived, and methods for applying the optimal combination of loss function, neural network, and data augmentation method for optimizing the performance of these technologies were presented. To optimize image segmentation performance in X-ray image datasets and ultrasound image datasets, the use of dice Loss or Tversky loss functions is recommended, and using UNet for filter-based data augmentation yielded optimal performance. In addition, in order to optimize the image classification performance in the pathology image data set, using ResNet50 for filter-based data propagation was able to produce optimal performance. Above all, selecting a data augmentation method that can compensate for the characteristics of the medical image dataset, selecting a neural network suitable for this, and selecting the corresponding loss function yielded optimal performance.

## Figures and Tables

**Table 1:** Image Recognition Technique and dataset for each medical images.

	Diagnosis	Diagnosis	Recognition	Evaluation
	Target	Accuracy	Technology	Dataset
X-ray	Microcalcification,	Medium	Segmentation	CBIS-DDSM
	Tumor Location			[17]
Ultrasound	Palable Mass,	Low	Segmentation	BUI [18]
	Tumor Location			
Histopathology	Breast Cancer	High	Classification	BHI [19]

**Table 2:** Applied data argumentation techniques.

Geometric	Rotation, Vertical Flip, Horizontal Flip, Grid Distortion
Filter	CLAHE, Gaussian, Equalization, Fancy PCA

**Table 3:** The performance according to loss functions in basic X-ray dataset.

Model	UNet			ResUNet++			DeepLabv3		
Loss	BC	DICE	Tversky	BC	DICE	Tversky	BC	DICE	Tversky
Metric	IOU	IOU	IOU	IOU	IOU	IOU	IOU	IOU	IOU
Vanilla	0.56	0.62	0.63	0.54	0.63	0.63	0.52	0.62	0.63
Metric	Dice	Dice	Dice	Dice	Dice	Dice	Dice	Dice	Dice
Vanilla	0.39	0.41	0.43	0.41	0.42	0.44	0.36	0.39	0.42

**Table 4:** The performance according to various data augmentation techniques in augmented X-ray datasets.

Model	UNet		ResUNet++		DeepLabv3	
Loss	DICE	Tversky	DICE	Tversky	DICE	Tversky
Metric	IOU	IOU	IOU	IOU	IOU	IOU
Vanilla	0.624	0.634	0.631	0.639	0.619	0.631
Filter	0.819	0.832	0.777	0.745	0.786	0.809
Geometric	0.714	0.711	0.701	0.685	0.691	0.691
All	0.771	0.774	0.736	0.724	0.737	0.754
Metric	Dice	Dice	Dice	Dice	Dice	Dice
Vanilla	0.411	0.438	0.421	0.441	0.392	0.417
Filter	0.786	0.808	0.712	0.658	0.729	0.765
Geometric	0.608	0.604	0.506	0.548	0.557	0.557
All	0.704	0.708	0.612	0.612	0.639	0.672

**Table 5:** The average time taken to segment a X-ray image.

Model	UNet	ResUNet++	DeepLabv3
latency	13	18	19

**Table 6:** The performance according to loss functions in basic Ultrasound dataset.

Model	UNet			ResUNet++			DeepLabv3		
Loss	BC	DICE	Tversky	BC	DICE	Tversky	BC	DICE	Tversky
Metric	IOU	IOU	IOU	IOU	IOU	IOU	IOU	IOU	IOU
Vanilla	0.75	0.76	0.79	0.67	0.78	0.75	0.75	0.79	0.79
Metric	Dice	Dice	Dice	Dice	Dice	Dice	Dice	Dice	Dice
Vanilla	0.71	0.72	0.76	0.61	0.74	0.70	0.73	0.77	0.76

**Table 7:** The performance according to various data augmentation techniques in augmented Ultrasound datasets.

Model	UNet		ResUNet++		DeepLabv3	
Loss	DICE	Tversky	DICE	Tversky	DICE	Tversky
Metric	IOU	IOU	IOU	IOU	IOU	IOU
Vanilla	0.758	0.789	0.778	0.752	0.793	0.792
Filter	0.984	0.980	0.962	0.962	0.980	0.978
Geometric	0.816	0.836	0.833	0.812	0.820	0.812
All	0.906	0.902	0.877	0.875	0.890	0.893
Metric	Dice	Dice	Dice	Dice	Dice	Dice
Vanilla	0.716	0.764	0.742	0.704	0.764	0.764
Filter	0.988	0.986	0.965	0.964	0.983	0.982
Geometric	0.799	0.800	0.808	0.789	0.799	0.789
All	0.905	0.902	0.870	0.872	0.885	0.887

**Table 8:** The average time taken to segment a Ultrasound image.

Model	UNet	ResUNet++	DeepLabv3
latency	13	18	19

**Table 9:** ACC and AUC of Histopathology according to Data Augmentation Techniques.

Model	VGG19		Dense121		ResNet50		EfficientNet	
metric	ACC	AUC	ACC	AUC	ACC	AUC	ACC	AUC
Vanilla	0.79	0.84	0.83	0.89	0.84	0.92	0.83	0.91
Mixup	0.84	0.87	0.85	0.92	0.91	0.96	0.83	0.92
CutMix	0.87	0.94	0.90	0.96	0.94	0.96	0.94	0.91
Filter	0.92	0.93	0.94	0.98	0.97	0.98	0.94	0.96
Geometric	0.82	0.87	0.88	0.94	0.90	0.95	0.85	0.94
Filter+CutMix	0.94	0.95	0.96	0.97	0.98	0.99	0.93	0.98

**Table 10:** SEN and SEP of Histopathology according to Data Augmentation Techniques.

Model	VGG19		Dense121		ResNet50		EfficientNet	
metric	SEN	SPE	SEN	SPE	SEN	SPE	SEN	SPE
Vanilla	0.78	0.78	0.82	0.82	0.84	0.84	0.83	0.88
Mixup	0.82	0.81	0.85	0.88	0.89	0.89	0.84	0.84
CutMix	0.88	0.88	0.90	0.90	0.89	0.89	0.85	0.85
Filter	0.85	0.86	0.93	0.93	0.97	0.97	0.91	0.91
Geometric	0.79	0.79	0.87	0.87	0.87	0.89	0.87	0.88
Filter+CutMix	0.89	0.89	0.91	0.91	0.98	0.98	0.93	0.93

**Table 11:** The average time taken to classify a pathology image.

Model	VGGNet19	ResNet50	DenseNet121	EfficientNet
latency	10	19	32	61
